# Biliary Amino Acids and Telocytes in Gallstone Disease

**DOI:** 10.3390/metabo13060753

**Published:** 2023-06-14

**Authors:** Jolanta Bugajska, Joanna Berska, Artur Pasternak, Krystyna Sztefko

**Affiliations:** 1 Department of Clinical Biochemistry, Institute of Pediatrics, Jagiellonian University Medical College, Wielicka St. 265, 30-663 Krakow, Poland; joanna.berska@uj.edu.pl (J.B.); krystyna.sztefko@uj.edu.pl (K.S.); 2 Department of Anatomy, Jagiellonian University Medical College, 12th Kopernika St., 31-034 Krakow, Poland; artur.pasternak@uj.edu.pl

**Keywords:** amino acids, bile acids, fatty acids, gallstone disease, telocytes

## Abstract

The role of amino acids in cholesterol gallstone formation is not known. Therefore, the aim of the study was to determine the amino acid profile in the bile of patients with and without cholecystolithiasis in relation to bile lithogenicity and telocyte numbers within the gallbladder wall. The study included 23 patients with cholecystolithiasis and 12 gallstone-free controls. The levels of free amino acids in the bile were measured, and telocytes were identified and quantified in the gallbladder muscle wall. The mean values of valine, isoleucine, threonine, methionine, phenylalanine, tyrosine, glutamic acid, serine alanine, proline and cystine were significantly higher in the study group than in the controls (*p* from 0.0456 to 0.000005), and the mean value of cystine was significantly lower in patients with gallstone disease than in the controls (*p* = 0.0033). The relationship between some of the amino acids, namely alanine, glutamic acid, proline, cholesterol saturation index (CSI) and the number of telocytes was significant (r = 0.5374, *p* = 0.0051; r = 0.5519, *p* = 0.0036; and r = 0.5231, *p* = 0.0071, respectively). The present study indicates a potential relationship between the altered amino acid composition of bile and the reduced number of telocytes in the gallbladder muscle wall in cholelithiasis.

## 1. Introduction

Gallstone disease (GD) constitutes a significant health problem in developed societies, affecting from 5% to 25% of the adult population [1,2]. Bile is produced by the hepatocytes and transferred to the gallbladder via the bile duct system. The main components of gallbladder bile are bile salts (60%), and about 20% bilirubin, cholesterol, fatty acids, and phospholipids [3]. Proteins represent only 5% of the biliary constituents by dry weight. Cholic acid and chenodeoxycholic acid are primary 24-carbon-atom bile acids (BAs) synthesized in hepatocytes from cholesterol.

Before excretion into the bile, primary BAs conjugate within hepatocytes. Conjugation of the terminal side-chain carboxylic acid with glycine or taurine is performed by the enzyme bile acid CoA: amino acid N-acyltransferase (BAAT) [4]. The primary BAs are converted to secondary BAs in the large bowel. In humans, deoxycholic acid, ursodeoxycholic acid, lithocholic acid, glycodeoxycholic acid and taurodeoxycholic acid belong to the secondary BAs [5].

Cholelithiasis can be caused by physiological, biophysical, cellular, molecular, and genetic factors. Mutations in the hepatic cholesterol transporter ABCG8 confer most of the genetic risk of developing gallstones [6]. An excessive concentration of cholesterol in bile associated e.g., with a high-fat diet, overproduction of mucin and disturbances in the motility of the gallbladder are known factors predisposing someone to the development of gallstone disease [7]. Moreover, bile cholesterol supersaturation may be caused by hepatic cholesterol hypersecretion, diminished hepatic secretion of biliary bile acids with relatively normal hepatic cholesterol secretion, reduced hepatic phospholipids secretion with relatively normal hepatic cholesterol secretion and a combination of hypersecretion of biliary cholesterol and hyposecretion of the solubilizing lipids [8]. Bile lithogenicity is expressed as a cholesterol saturation index (CSI) [9,10]. On the other hand, patients with cholelithiasis have reduced telocyte (TCs) density in the gallbladder wall, which can cause gallbladder hypomotility and precipitation of the cholesterol crystals from lithogenic bile [11]. Knowledge about gallbladder motility has been linked to a novel population of cells called telocytes (TCs). TCs are an interstitial (stromal) cell type already identified in many tissues and organs, including the human gallbladder. These cells are characterized by a small cell body (9–15 μm) and a variable number (one to five) of extremely long and thin telopodes (Tps), with alternating regions of podomers (~80 nm) and podoms (250–300 nm). Telopodes are interconnected by homo- and heterocellular junctions, and form three-dimensional networks [12,13]. Previously, we reported a significant decrease in c-Kit positive TCs density in the gallbladder wall in patients suffering from cholelithiasis [14].

The plasma profile of amino acids is altered in gallstone disease [15], but little information is available concerning the presence and significance of amino acids in bile and their potential role in bile lithogenicity. The specific treatment for cholelithiasis and its potential complications has been known, but a better understanding of factors for the development of cholesterol gallstones might serve to individualize the treatment of patients. The purpose of the study was to evaluate the amino acid profile in the bile of patients with and without cholecystolithiasis in relation to bile lithogenicity and telocyte numbers within the gallbladder wall.

## 2. Materials and Methods

The study included 23 patients (19/4, F/M) with symptomatic gallstone disease presenting with mild, recurrent episodes of biliary colic. None of these patients had associated choledocholithiasis or acute cholecystitis. Gallstones were visualized in the gallbladder on ultrasound examination before surgery. The control group consisted of 12 consecutive patients (6/6, F/M) who were electively treated for pancreatic head tumours and had no pre- or intraoperative signs of cholelithiasis and jaundice. During the pancreatoduodenectomy procedure due to a pancreatic head tumour, the gallbladder was routinely excised—however, only those gallbladders that did not have any concrements (gallstones) in the lumen and were not infiltrated by the cancer process were taken into account in the group. The mean age (SD) of patients with gallstone disease was 51.1 (14.6) years, and 62.7 (9.0) years in the control group (*p* = 0.009). Preoperative serum bilirubin concentrations were normal in all patients. Bile specimens were obtained from all subjects during surgery, which was performed in the First Department of General, Oncological and Gastrointestinal Surgery at the Jagiellonian University Medical College.

Aliquots of bile samples were stored at −70 °C before analysis. Cholesterol, phospholipid, and total bile acid concentrations, as well as the percentage of individual fatty acids (FAs) of the phospholipid (PL) fraction, individual free amino acids (AAs), and individual bile acids (BAs) in the bile samples were determined. The enzymatic methods were used to measure concentrations of cholesterol (Randox Laboratories Ltd., Crumlin, UK) and phospholipid (Wako Chemicals, Neuss, Germany) [12]. Measurement of the individual fatty acids of the phospholipid fraction in the bile was performed using gas chromatography with the flame ionization detector (Agilent Technologies 6890, Santa Clara, CA, USA). Bile levels of saturated fatty acids (SFAs): lauric (C12), myristic (C14), palmitic (C16), stearic (C18); monounsaturated fatty acids (MUFAs): palmitoleic (C16:1 n-7), oleic (C18:1 n-9) and polyunsaturated fatty acids: linoleic (C18:2 n-6), alfa-linolenic (C18:3 n-3), eicosadienoic (C20:2 n-6), arachidonic (C20:4 n-6), eicosapentanoic (C20:5 n-3), docosahexaenoic (C22:6 n-3) and the sum of arachidic acid (C20) and gamma-linolenic acid (C18:3 n-6) of the phospholipid fraction were measured [16]. Individual bile acids were measured using reverse-phase high-performance liquid chromatography (HPLC) with UV-VIS detection (Waters, USA). Bile levels of glycocholic acid (GCA), taurocholic acid (TCA), glycochenodeoxycholic acid (GCDCA), glycodeoxycholic acid (GDCA), taurochenodeoxycholic acid (TCDCA) and taurodeoxycholic acid (TDCA) were measured [12]. The determination of the free amino acid (AAs) profile in the bile samples was performed using the liquid chromatography–tandem mass spectrometry method (LC-MS/MS; 1260 Infinity II, 6460 QTRAP; Agilent Technologies, Waldbronn, Germany) with a quantitative amino acids analysis kit (Jasem, Istanbul, Turkey). The following amino acids were determined: tryptophan, taurine, phenylalanine, tyrosine, leucine, isoleucine, methionine, valine, glutamic acid, aspartic acid, α-aminobutyric acid, threonine, serine, alanine, glycine, asparagine, proline, citrulline, cystine, arginine, histidine, ornithine, lysine and 3-methyl-histidine. The level of fatty acids, amino acids and bile acids in the bile was expressed as the percentage of the total amounts of the respective groups of compounds. The cholesterol saturation index was calculated by dividing the cholesterol concentration by the maximum cholesterol solubility according to Carey and Small [9], and corrected for the total lipid content of each individual bile [10]. Bile samples were considered supersaturated if the CSI value was equal to 1 or more [8].

Tissue samples from cholecystectomy specimens were collected. The identification of telocytes in the gallbladder was performed using a double immunofluorescence technique. Quantitative analysis was carried out under fluorescence microscopy conjoined with image analysis [11].

The study was conducted according to the guidelines of the Declaration of Helsinki and approved by the Jagiellonian University Ethics Committee (Protocol No. 1072.6120.138.2021). Written consent was obtained from all patients. All methods performed in the study were conducted following all ethical and legal regulations.

Descriptive statistics (mean [SD], medians [interquartile ranges]) were used in the statistical assessment of the obtained results. The Statistica software version 13 (StatSoft, Kraków, Poland) was used to perform statistical analysis. To evaluate the distribution of continuous variables in terms of compliance with the normal distribution, the Shapiro–Wilk test was employed. To compare parameters between the study group and the control group for normally distributed continuous variables, the *t*-test was used, and in the case of non-normal distribution, the Mann–Whitney U test was used. Spearman’s correlation was used to examine relationships between fatty acids, amino acids, bile acids and both the cholesterol saturation index and the number of telocytes. The level of significance was set at a *p*-value of less than 0.05.

## 3. Results

No significant difference in mean concentrations of cholesterol, bile salts, and phospholipids was obtained between patients with gallstone disease and the controls. Additionally, no differences in the median values of CSI were noticed (*p* = 0.32), but a significantly lower mean [SD] number of telocytes in mm2 in the study group compared with the control group (26.5 [10.6] vs. 53.5 [15.0]) was observed.

Patients with gallstone disease had significantly higher mean values of the percentage of bile total fatty acid content of C18:2 (n-6) and C18:3 (n-3) (*p* = 0.008; *p* = 0.01, respectively), as compared to the controls. In contrast, the mean value of the percentage of bile total fatty acid content of C16 was significantly higher in the controls as compared to the study group (*p* = 0.04) (Table 1).

Significantly lower mean values for the percentage of glycocholic acid (*p* = 0.03) in the bile from the cholelithiatic gallbladder was observed, compared to the control group. In contrast, the mean values for the percentage of glycodeoxycholic acid and taurodeoxycholic acid were significantly higher in the study group as compared to the controls (*p* = 0.01; *p* = 0.03, respectively), (Table 2).

The mean values of valine, isoleucine, threonine, methionine, phenylalanine, tyrosine, glutamic acid, serine alanine, proline and cystine were significantly higher in the study group than in the controls (*p* from 0.045 to <0.001), but the mean value of cystine was significantly lower in patients with gallstone disease than in the controls (*p* = 0.003) (Table 3). No differences for other amino acid concentrations between these groups were noticed.

**Figure 1:**
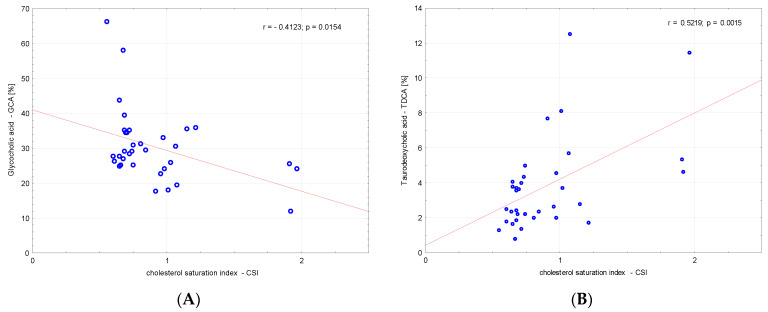
Correlations between (**A**) glycocholic acid—GCA, (**B**) TDCA—taurodeoxycholic acid and the cholesterol saturation index (CSI).

There was no correlation between amino acids and CSI and between fatty acids and CSI. A significant negative correlation between glycocholic acid (GCA) and CSI (r = −0.4123, *p* = 0.02) and a significant positive correlation between taurodeoxycholic acid (TDCA) and CSI (r = 0.5219, *p* = 0.002) was found (Figure 1).

**Figure 2:**
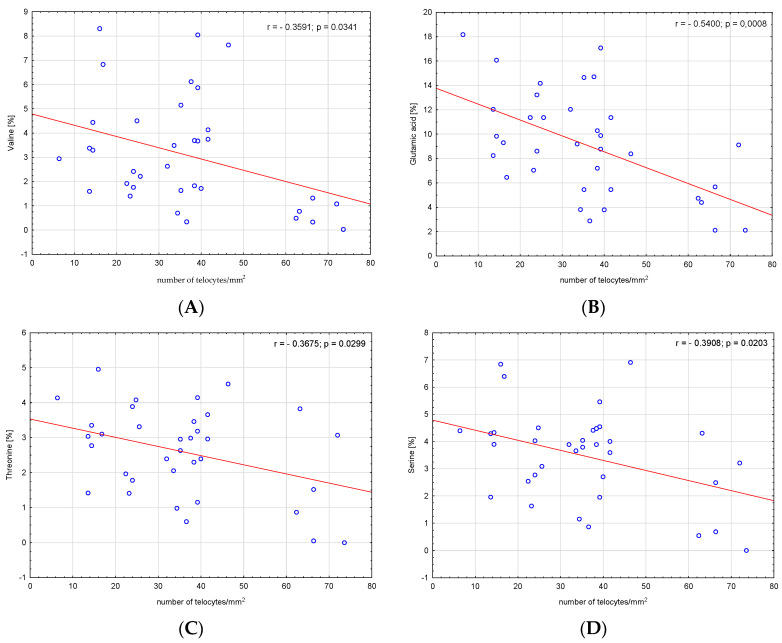

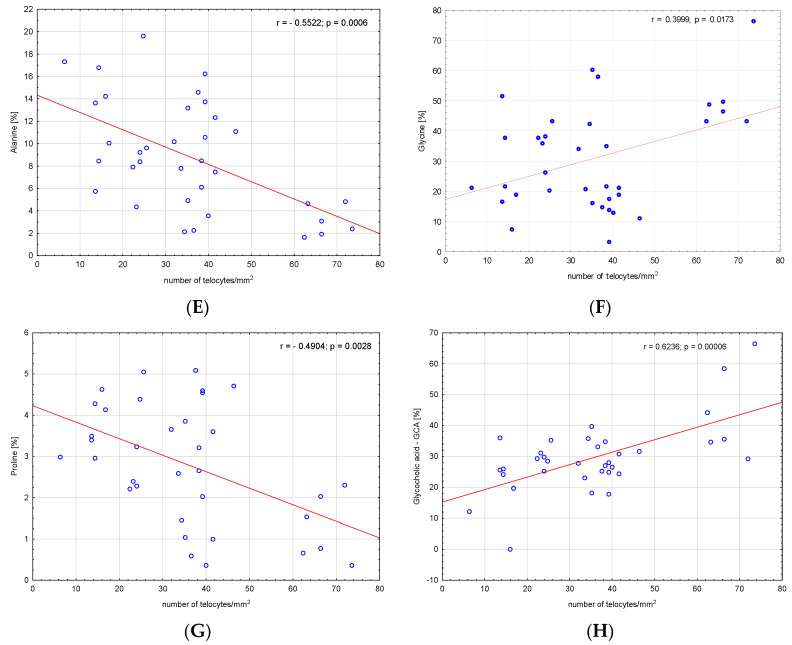
Correlations between (**A**) valine, (**B**) glutamic acid, (**C**) threonine, (**D**) serine, (**E**) alanine, (**F**) glycine, (**G**) proline, (**H**) glycocholic acid—GCA and the number of telocytes.

**Figure 3:**
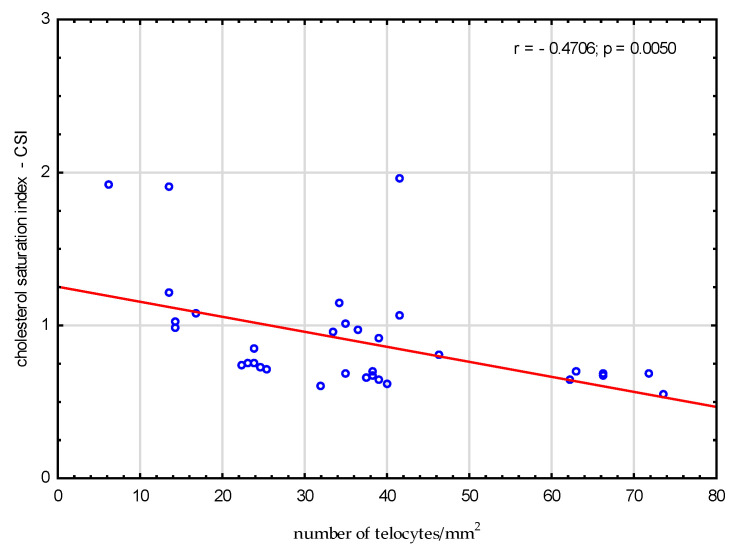
Correlations between cholesterol saturation index CSI and the number of telocytes.

We analysed the correlations between amino acids, fatty acids, bile acids and the number of telocytes. We found a significant negative correlation between valine, glutamic acid, threonine, serine, alanine, proline and number of telocytes (r = −0.3591, *p* = 0.03; r = −0.54, *p* < 0.001; r = −0.3675, *p* = 0.03; r = −0.3908, *p* = 0.02; r = −0.5522, *p* < 0.001; and r = −0.4904, *p* = 0.003, respectively), (Figure 2). Additionally, we found significant negative correlations between CSI and the number of telocytes (r = −0.4706, *p* = 0.005) (Figure 3). Glycine and glycocholic acid correlated significantly and positively with the number of telocytes (r = 0.3999, *p* = 0.02; and r = 0.6236, *p* < 0.001, respectively), (Figure 2).

**Figure 4:**
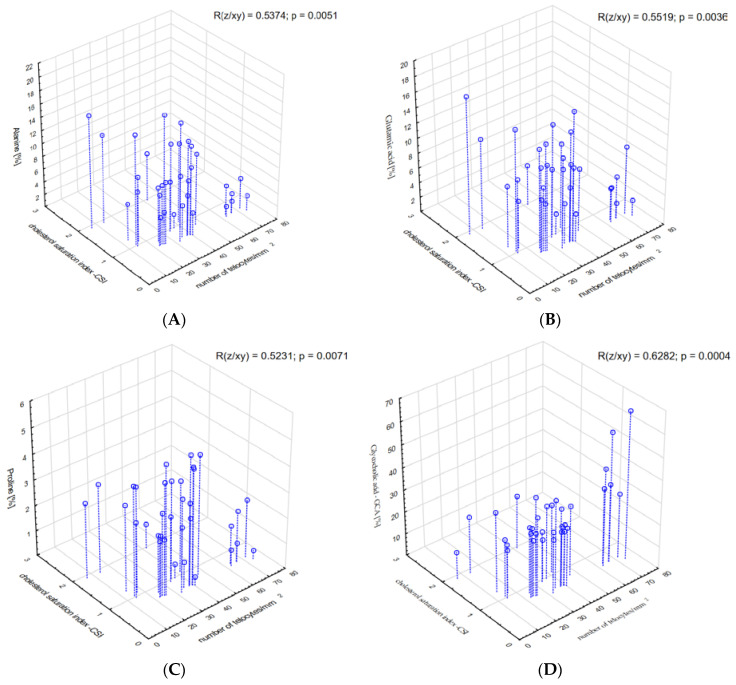
Correlations between (**A**) alanine, (**B**) glutamic acid, (**C**) proline, (**D**) glycocholic acid—GCA and the cholesterol saturation index and the number of telocytes.

The relationship between some of amino acids, namely alanine, glutamic acid, proline, CSI and the number of telocytes was significant (R = 0.5374, *p* = 0.005; R = 0.5519, *p* = 0.004; and R = 0.5231, *p* = 0.007, respectively), (Figure 4A–C). A similar significant relationship between GCA and CSI and the number of telocytes was found (R = 0.6282, *p* < 0.001, respectively), (Figure 4D).

## 4. Discussion

Research on gallstone disease mainly concentrates on lipids and bile acids, and no information on protein and amino acids in bile can be found. The main proteins in bile are mucins. It was shown that mucus on the surfaces of cell membranes is important in the diagnosis, prognosis and monitoring of a variety of diseases [17]. Gallbladder mucin is the major secretory glycoprotein of the gallbladder epithelium. Mucins are produced by cholangiocytes, the dominant epithelial cell type in the gallbladder. Inoue et al. [18] demonstrated that cholesterol gallstone bile was characterized by high concentrations of prostaglandins. The synthesis and secretion of bile mucin are stimulated by prostaglandins [18]. It is known that prostaglandins are derived from arachidonic acid—C 20:4 (n-6). C 20:4 (n-6) originates from C18:2 (n-6). In our study, the level of C18:2 (n-6) in the phospholipid fraction of bile was higher in the patients with gallstone disease than in the control group. This could cause the increased synthesis of prostaglandins in patients with gallstone disease, and, in the end, the overproduction of mucin.

The overproduction of mucin is involved in gallstone formation. The mucus glycoprotein gel on the surface of the gallbladder mucosa may trap cholesterol microcrystals in the first stage of gallstone formation [19,20]. All of the mucins in the gallbladder are exposed constantly to bile, which is why the amino acid profile in bile may reflect the presence of mucins. An analysis of mucin genes revealed amino acid sequences of different mucins. The human MUC3 and MUC5B genes are highly expressed in the gallbladder epithelium, whereas a weak expression of MUC5AC has been detected in the gallbladder [21]. In the gallbladder, MUC6 had also been found. It is suggested that the primary function of MUC6 in the gallbladder is the protection of vulnerable epithelial surfaces from the damaging effects of the constant exposure to bile. MUC6 is not capable of gel formation, which means that it differs markedly from the other human mucins, i.e., MUC2 and MUC5B, which are cysteine-rich. The translated sequence of the MUC6 is rich in threonine, serine, and proline (15.5, 24.7 and 11.4%, respectively), with cysteine residues (3.0%) [22,23]. Lee et al. [24] found altered mucin gene expression in gallbladders with cholesterol stones and calcium bilirubinate stones. They showed a stronger and more extensive expression of mRNA of MUC1, MUC3, MUC5B and MUC6 in gallbladders with stones than in gallbladders without stones. MUC2 and MUC4 expression was found only in gallbladders with stones [24]. MUC4 may be positively associated with the calcification of cholesterol gallstones [25]. Mucins are proteins that are substituted with oligosaccharides in O-linked serine or threonine residues. Mucin core proteins typically contain a series of tandem repeat domains enriched in serine, threonine and proline residues [26]. MUC2 is extremely rich in threonine and proline (55–60 and 22%, respectively), whereas MUC3 is a threonine- and serine-rich peptide (29–41 and 25–29%, respectively) [27]. In the present study, the mean value of threonine, proline and serine in the bile in the patients with gallstone disease was significantly higher than in the control group, probably due to the overproduction of mucin. Additionally, a significant negative correlation between the values of serine, threonine, proline and the number of telocytes in the wall of the gallbladder corpus was found. In addition, the value of proline was negatively correlated with CSI and the number of telocytes. It may indicate the overproduction of mucin, which may cause telocyte injury.

Cholesterol gallstones contain calcium and pigment at their centres. Additional small acidic proteins seem likely to play a role in the pathogenesis of cholesterol gallstones. This is known as a phenomenon of the gallstone, because such proteins are also important regulators of other biomineralization systems such as bone and dentin, as well as in urinary and pancreatic calculi. The proteins from the cholesterol gallstones are mainly small in size and are highly acidic, due to a high content of acidic amino acids (aspartic acid and glutamic acid) [28]. In the present study, the mean values of these two acidic amino acids in bile were higher in patients with cholelithiasis (13.48%) than in the controls (6.75%). This was mainly due to the higher value of glutamic acid in the study groups than in the controls (*p* < 0.001). Basic amino acids (lysine, arginine, histidine) did not show a difference between the study groups (7.25%) and the controls (7.04%). This is in agreement with the results obtained by Shimizu et al. [28], who analysed the amino acid composition of cholesterol gallstones. They showed a high content (mean 21.4 residue%) of the acidic amino acids (aspartic acid and glutamic acid) and low content (mean 10.1 residue%) of basic amino acids (lysine, arginine, histidine). The cited authors also found high percentages of aliphatic amino acids (glycine, alanine, valine, isoleucine and leucine), as well as the hydroxylated residues, serine and threonine, in cholesterol gallstones [28]. This is in agreement with the data of the present study. It was found that in patients with gallstone disease the mean values of alanine, valine, isoleucine, leucine, serine and threonine were significantly higher as compared to the controls. Additionally, we found a significant negative correlation between the levels of glutamic acid, alanine, valine, serine and threonine, and the number of telocytes in the wall of the gallbladder body. In addition, the levels of alanine and glutamic acid were negatively correlated with the cholesterol saturation index and the number of telocytes in the wall of the gallbladder body. The increased amount of acidic amino acids in bile may cause the formation of stones.

In the gallbladder bile of patients without gallbladder pathology, lower total glutathione and lower oxidised glutathione levels compared with those of patients with cholecystitis with/or without cholecystolithiasis were already proven [29]. It is well known that glutathione contains amino acids: glycine, glutamate, and cysteine, with cysteine being the limiting compound for glutathione synthesis in normal tissues. The main form of extracellular cysteine, and a major form in which cysteine is taken by tissues, is cystine. Extracellular cystine is transported into the cell through a cystine/glutamate antiporter, while glutamate is exported. Cystine, after entering the cells, is converted to cysteine, which is subsequently used for the synthesis of proteins, glutathione, and other sulfur-containing molecules [30]. The major use of cysteine is the production of the antioxidant glutathione. This tripeptide exists in a reduced (active) and oxidised form. One of the most widely recognised functions of reduced glutathione is the protection of cells and tissues against toxic compounds, of both endogenous and exogenous origin [29,31]. In the present study, in patients with gallstone disease the mean values of glycine and cystine were significantly lower as compared to the controls. This may be due to the production of glutathione in response to the presence of stones in the gallbladder.

The role of bile acids in gallstone disease is well known. Wu L et al. [32] showed a decreased content of GCA in plasma in the cholecystolithiasis group compared to the control group; in addition, they showed that GCA plays a key role in the health of the biliary system. Our study simply confirms this results. The levels of GCA were negatively correlated with the cholesterol saturation index and negatively with the number of telocytes. These results suggest that GCAs are protective of TCs. Additionally, the levels of TDCA (secondary BAs) were positively correlated with the CSI. Primary and secondary BAs are players in the development of gallstone disease. However, the significant correlation between some amino acids (alanine, glutamic acid, proline) and CSI and telocytes may indicate separate amino acid participation in gallstone formation.

There are two limitations to the study. First, significant differences in age between the controls and the patients with gallstone disease call into question the results of the study. However, by checking the relation between amino acid value and patient age, no correlation has been found for any amino acid, so differences seen in amino acids in bile are the real finding not related to age. Second, there were a small number of patients in the control group. We cannot confirm or exclude the role of amino acids in stone formation, and further research on gallstone disease should take into account the amino acid profile in bile.

## 5. Conclusions

The present study indicates a potential relationship between altered amino acids and bile acid composition of bile and the reduced TC count in cholelithiasis.

## Figures and Tables

**Table 1:** The mean (SD) or median (interquartile range) values of bile fatty acids of phospholipids fraction in the control group and patients with gallstone disease (study group), expressed as the percentage of total fatty acid content.

Fatty Acid	Control Group	Study Group	
	Percentage of Total FAs Mean (SD) or Median (Interquartile Range)		*p*
SFAs			
C 12	0.03 (0.02–0.04)	0.03 (0.02–0.04)	0.72
C 14	0.45 (0.20)	0.51 (0.18)	0.43
C 16	42.5 (40.9–43.5)	40.4 (40.0–41.2)	0.04
C 18	5.91 (5.04–6.57)	6.13 (5.43–6.80)	0.61
MUFAs			
C 16:1 (n-7)	2.42 (1.22)	2.10 (0.97)	0.41
C 18:1 (n-9)	12.2 (10.3–13.6)	11.2 (9.9–13.0)	0.39
PUFAs			
C 18:2 (n-6)	23.4 (22.2–25.5)	28.8 (25.8–31.2)	0.008
C 18:3 (n-3)	0.39 (0.20)	0.58 (0.21)	0.01
C 20:2 (n-6)	0.14 (0.10–0.15)	0.16 (0.13–0.20)	0.15
C 20:4 (n-6)	6.77 (3.64)	6.54 (1.28)	0.78
C 20:5 (n-3)	1.26 (1.03)	1.15 (0.61)	0.68
C 22:6 (n-3)	2.26 (0.79)	1.97 (0.65)	0.26
C 20 + C 18:3 (n-6)	0.25 (0.15–0.50)	0.28 (0.20–0.40)	0.77

SFAs—saturated fatty acids; MUFAs—monounsaturated fatty acids; PUFAs—polyunsaturated fatty acids

**Table 2:** The mean (SD) or median (interquartile range) values of bile acids in bile in the control group and for patients with gallstone disease (study group), expressed as the percentage of total bile acid content.

Bile Acids	Control Group	Study Group	
	Percentage of Total BAs Mean (SD) or Median (Interquartile Range)		*p*
Primary bile acids			
GCA	33.8 (27.8–39.9)	27.0 (24.2–30.8)	0.03
TCA	10.4 (6.42–19.1)	7.57 (5.59–10.7)	0.22
GCDCA	19.9 (16.5–29.6)	25.8 (22.5–29.7)	0.16
TCDCA	10.8 (7.0)	8.6 (4.6)	0.22
Secondary bile acids			
GDCA	13.7 (9.9)	23.4 (8.9)	0.001
TDCA	2.02 (1.74–3.21)	3.9 (2.4–5.0)	0.03

GCA—glycocholic acid; TCA—taurocholic acid; GCDCA—glycochenodeoxycholic acid; TCDCA—taurochenodeoxycholic acid; GDCA—glycodeoxycholic acid; TDCA—taurodeoxycholic acid.

**Table 3:** The mean (SD) or median (interquartile range) values of bile amino acids in the control group and for patients with gallstone disease (study group), expressed as the percentage of total amino acid content.

Amino Acids	Control Group	Study Group	
	Percentage of Total AAs Mean (SD) or Median (Interquartile Range)		*p*
Valine	0.93 (0.41–2.92)	3.38 (1.92–4.51)	0.01
Isoleucine	0.89 (0.46–2.65)	2.01 (1.50–2.56)	0.03
Leucine	0.66 (0.45–4.97)	2.68 (1.81–3.85)	0.06
Threonine	1.89 (1.56)	2.97 (0.92)	0.01
Methionine	0.02 (0.01–0.04)	0.05 (0.03–0.19)	0.02
Phenylalanine	0.30 (0.11–1.16)	1.09 (0.68–1.50)	0.045
Lysine	4.11 (2.87)	4.98 (2.01)	0.30
Tryptophan	0.38 (0.20)	0.48 (0.24)	0.18
Histidine	0.78 (0.49–1.13)	0.67 (0.33–0.94)	0.46
Arginine	2.15 (1.70–3.17)	1.60 (1.11–2.27)	0.10
Tyrosine	0.45 (0.32–0.90)	1.09 (0.74–1.71)	0.01
Aspartic acid	1.65 (0.85–1.89)	2.08 (1.45–2.35)	0.09
Glutamic acid	5.10 (2.48)	11.4 (3.45)	<0.001
Serine	2.37 (1.96)	4.03 (1.20)	0.004
Asparagine	0.08 (0.00–0.36)	0.16 (0.05–0.43)	0.45
Glycine	38.8 (20.4)	27.0 (13.8)	0.05
Taurine	15.2 (9.7–25.4)	10.0 (6.09–19.0)	0.14
Citrulline	0.01 (0.00–0.19)	0.10 (0.01–0.19)	0.13
Alanine	3.3 (2.18–6.14)	10.0 (7.9–13.7)	<0.001
Proline	1.22 (0.62–2.02)	3.49 (2.65–4.39)	<0.001
Ornithine	3.31 (2.10–4.62)	3.04 (2.36–4.16)	0.79
3-methyl-histidine	0.05 (0.02–0.07)	0.03 (0.03–0.06)	0.93
Cystine	2.86 (1.84–5.96)	1.04 (0.59–1.97)	0.003
α-Aminobutyric acid	0.32 (0.20–0.39)	0.23 (0.12–0.35)	0.22

## Data Availability

Due to privacy, the data presented in this study are available from the corresponding author upon reasonable request.
